# Outcome prediction in sepsis: Speckle tracking echocardiography based assessment of myocardial function

**DOI:** 10.1186/cc13987

**Published:** 2014-07-11

**Authors:** Sam R Orde, Juan N Pulido, Mitsuru Masaki, Shane Gillespie, Jocelyn N Spoon, Garvan C Kane, Jae K Oh

**Affiliations:** 1 Division of Cardiovascular Diseases, Mayo Clinic, 200 First St SW, Rochester, MN 55905, USA; 2 Department of Intensive Care, Nepean Hospital, Sydney, Australia; 3 Department of Anesthesiology, Mayo Clinic, 200 First St SW, Rochester, MN 55905, USA; 4 Hyogo College of Medicine, Nishinomiya, Hyogo, Japan

## Abstract

**Introduction:**

Speckle tracking echocardiography (STE) is a relatively novel and sensitive method for assessing ventricular function and may unmask myocardial dysfunction not appreciated with conventional echocardiography. The association of ventricular dysfunction and prognosis in sepsis is unclear. We sought to evaluate frequency and prognostic value of biventricular function, assessed by STE in patients with severe sepsis or septic shock.

**Methods:**

Over an eighteen-month period, sixty patients were prospectively imaged by transthoracic echocardiography within 24 hours of meeting severe sepsis criteria. Myocardial function assessment included conventional measures and STE. Association with mortality was assessed over 12 months.

**Results:**

Mortality was 33% at 30 days (n = 20) and 48% at 6 months (n = 29). 32% of patients had right ventricle (RV) dysfunction based on conventional assessment compared to 72% assessed with STE. 33% of patients had left ventricle (LV) dysfunction based on ejection fraction compared to 69% assessed with STE. RV free wall longitudinal strain was moderately associated with six-month mortality (OR 1.1, 95% confidence interval, CI, 1.02-1.26, p = 0.02, area under the curve, AUC, 0.68). No other conventional echocardiography or STE method was associated with survival. After adjustment (for example, for mechanical ventilation) severe RV free wall longitudinal strain impairment remained associated with six-month mortality.

**Conclusion:**

STE may unmask systolic dysfunction not seen with conventional echocardiography. RV dysfunction unmasked by STE, especially when severe, was associated with high mortality in patients with severe sepsis or septic shock. LV dysfunction was not associated with survival outcomes.

## Introduction

Characterized by hemodynamic distress, severe sepsis is frequently associated with cardiopulmonary dysfunction driven by a cascade of cellular and molecular processes [1]. Myocardial dysfunction occurs frequently, early and involves both ventricles [2,3]. Whether myocardial dysfunction is related to outcome is unclear and may in part be related to the definition and modality of assessment. Echocardiography plays a crucial role in the noninvasive assessment of cardiac function in the ICU [4], but the optimal measure of ventricular dysfunction, particularly for the right ventricle (RV), has not been well established. Interpretation of changes in volumetric measures such as fractional area change (FAC) or ejection fraction can be affected by swings in volume status and loading conditions, frequent features in sepsis, and may not reflect well underlying contractility. Furthermore, such measures may lack sensitivity.

Two-dimensional speckle tracking echocardiography (STE) has emerged as an angle-independent technique for quantifying systolic function by assessing myocardial deformation [5,6]: strain and strain/time (strain rate). STE has been shown to be a feasible and sensitive quantitative technology for assessing ventricular contractile function in a variety of different cardiovascular diseases such as chemotherapy-induced cardiotoxicity [7], amyloidosis [8,9], preeclampsia [10] and in a pediatric cohort with severe sepsis [11]. The main focus of STE has been left ventricle (LV) global longitudinal strain (GLS), reflecting the function of the subendocardial myocardial fibers, which are oriented longitudinally. These fibers are especially sensitive to ischemia and increased wall stress [12]. STE has potentially even greater applicability to the quantitative assessment of RV function. Distinct from the LV, the RV has a preponderance of longitudinal fibers and therefore a greater proportion of contractility of the RV occurs from base to apex [13]. Longitudinal STE is hence well poised to act as a robust measure of RV contractility: RV free wall strain and RV free wall strain rate.

The objectives of this study were to assess: the prevalence of RV and LV dysfunction in severe sepsis and septic shock assessed with STE; factors related to RV and LV longitudinal strain dysfunction; and whether myocardial dysfunction assessed by STE is associated with mortality at 30 days and 6 months.

## Methods

We prospectively studied 60 adult patients (>18 years) with severe sepsis or septic shock admitted over an 18-month period at St. Mary’s Hospital, Rochester, MN, USA. The study was approved by the Mayo Institutional Review Board and written consent was obtained from all patients or authorized representatives (next of kin) before enrollment. Individuals were included by American College of Chest Physicians criteria for severe sepsis or septic shock [14]. Sepsis was defined by two or more criteria: temperature >38°C or <36°C, heart rate >90 beats/minute, respiratory rate >20 breaths/minute or arterial partial pressure of carbon dioxide <32 Torr (<4.3 kPa), white cell count >12,000 cells/mm^3^, <4,000 cells/mm^3^, or >10% immature (band) forms. Severe sepsis was defined as sepsis associated with organ dysfunction (Sequential Organ Failure Assessment (SOFA) score ≥2), hypoperfusion (lactate >2.3 mmol/dl, our institutional high normal value) or hypotension (systolic blood pressure <90 mmHg or decreased 40 mmHg below baseline). Severe sepsis with hypotension resistant to intravenous fluids was considered septic shock. Exclusion criteria were supraventricular tachyarrhythmias, pregnancy, congenital heart disease, cardiomyopathy, moderate or severe valvular disease and valvular prosthesis and insufficient image quality for STE.

### Echocardiography

Transthoracic echocardiography was performed within 24 hours of meeting sepsis criteria with a Vivid 7 echocardiography machine (GE Medical Systems, Milwaukee, WI, USA) by research sonographers or research fellows fully trained in echocardiography and strain imaging. A comprehensive echocardiogram was performed according to American Society of Echocardiography guidelines [15]. Physiologic parameters were recorded at the time of echocardiography. LV systolic dysfunction was classified by ejection fraction: present (<55%) or absent (>55%), and mild (45 to 54%), moderate (30 to 44%) or severe (<30%). The RV was assessed at end expiration in a multimodal fashion as per American Society of Echocardiography guidelines (tricuspid annular plane systolic excursion, lateral tricuspid annular velocity, RV wall motion, FAC) [16] and was classified as normal, mild, moderate or severe dysfunction. Parameters for abnormal RV systolic function were defined as tricuspid annular plane systolic excursion <16 mm, FAC <35%, Tricuspid valve systolic motion velocity <10 cm/second or reduced RV wall motion. For severe dysfunction, RV wall motion was severely reduced and/or FAC was <17% [16]. RV size was measured – basal, mid and longitudinal dimensions (abnormal above 42 mm, 35 mm and 86 mm respectively) – and compared with the LV size. Images were analyzed by physicians fully trained in echocardiography (MM, JKO, JNP).

### Speckle tracking echocardiography analysis

**Figure 1:**
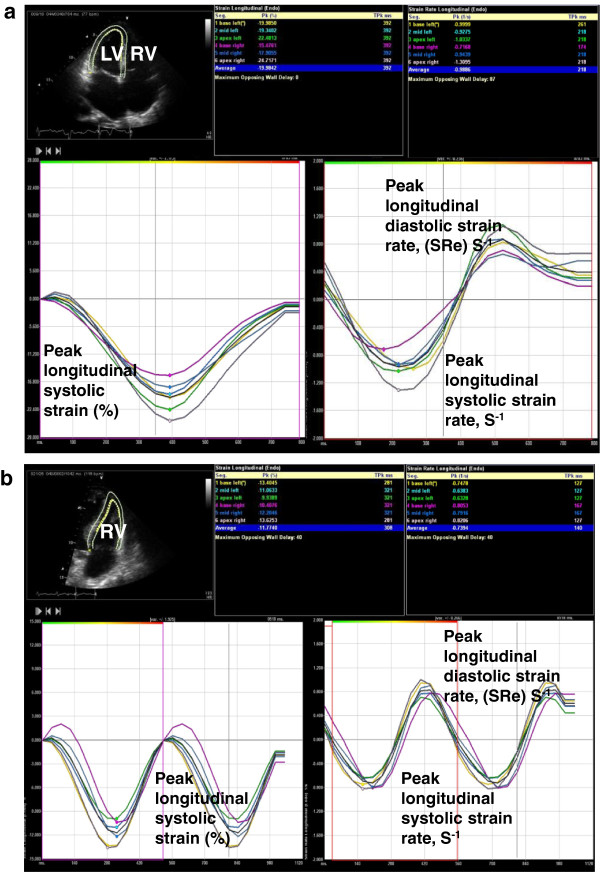
**Longitudinal strain and strain rate curves. (a)** Representative recording for apical four-chamber longitudinal strain and strain rate curves for a patient with normal left ventricle (LV) systolic function. Echo image displayed in Mayo format: left, LV; right, right ventricle (RV). Negative strain values indicate tissue contraction. Strain rate determined by change in strain over time. **(b)** Representative recording for apical four-chamber RV longitudinal strain and strain rate curves for a patient with abnormal RV systolic function. Echo image displayed in Mayo format: left, LV; right, RV. RV free wall longitudinal strain determined by the average of base, mid and apical free wall segments.

Three-beat two-dimensional digital clips were transferred to a Syngo Velocity Vector Imaging workstation (Siemens Medical Solutions USA Inc., Pleasanton, CA, USA) for STE analysis by SRO, who had performed more than 100 hours of analysis in STE prior to commencing the study. The endocardium was traced manually from the medial annulus with 7 to 15 points. LV values were averages of the 16 LV segments. If STE could not be calculated on one apical view, the LV was considered to have insufficient image quality. RV values were an average of the three free wall segments. Once accuracy of tracking was ensured, displacement, velocity, strain and strain rate curves were assessed for motion, smoothness, time to peak, delay and correlation (Figure 1a,b). The same cardiac cycle was chosen for STE values. All images were analyzed three times to ensure accuracy of results. Strain and strain rate are negative values; the more negative the value, the greater the degree of deformation and the better the function. Strain values were separated into normal (more negative than −21% for RV and more negative than −17% for LV), mild/moderately impaired (−21 to −13% for RV and −17 to −10% for LV) and severely abnormal (less negative than −13% for RV and less negative than −10% for LV). A consensus on normal values for strain of the RV and LV has yet to be defined primarily due to vendor differences in analysis methods [17]. The cutoff values chosen in this study are based on normal subjects at our institution [18] and on meta-analysis of normal subjects [19], and are similar to recent studies investigating LV ischemia [20] and pulmonary hypertension [21] as well as analysis of our sample group: receiver operating curve, interquartile range and logistic regression analysis.

### Statistical analysis

Statistical analysis was performed with JMP version 9.0.1 (SAS Institute Inc., Cary, NC, USA). Continuous variables are expressed as mean ± standard deviation or median with interquartile range and were analyzed between groups using analysis of variance. Categorical variables are expressed as the number and percentage with comparisons by Pearson’s chi-square analysis or Fisher’s exact test. All probability values are two-sided and of *P* ≤0.05 was considered significant. Univariate and multivariate logistic regression analyses were used to assess the association between risk factors and mortality. Discriminatory performance is assessed by odds ratio, 95% confidence interval and area under the receiver operating characteristic curve. Multivariate models were developed with stepwise inclusion and exclusion at a significance level of 0.1 and by consideration of variables that were clinically relevant.

## Results

Of 106 patients who were enrolled at our institution during the 18-month study period with severe sepsis or septic shock, 60 patients were included in our observational study. Of those excluded, 21 patients (20%) had supraventricular arrhythmia and 14 patients (13%) had insufficient image quality for STE analysis (10 of the 14 were mechanically ventilated). The mean age was 62 years (±15) with 50% female, 67% alive at 30 days (*n* = 40) and 52% alive at 6 months (*n* = 31) (Table 1). The SOFA score, arterial partial pressure of oxygen/fraction of inspired oxygen ratio, partial pressure of carbon dioxide and lactate levels were significantly worse in nonsurvivors at 30 days. The SOFA score was also significantly higher in nonsurvivors at 6 months. Thirty-nine patients (65%) were mechanically ventilated at the time of imaging; at 30 days a greater portion of these patients were alive (21 of 39 patients), but only 15 of the 39 were alive at 6 months. No difference was seen in comorbidities between the patient groups.

**Table 1:** Baseline physiological and clinical data with comparison for survival at 30 days and 6 months

**Characteristic**	**Baseline**	**30-day mortality**		**6-month mortality**	
		**Survivors**	**Nonsurvivors**	**Survivors**	**Nonsurvivors**
Mortality	60	40 (67%)	20 (33%)	31 (52%)	29 (48%)
Physiology					
Age (years)	62 ± 15	60 ± 16	65 ± 13	60 ± 17	65 ± 13
Female (%)	50	30	20	21.7	28.3
SOFA score	11 ± 4	10 ± 4*	13 ± 3*	10 ± 4*	12 ± 4*
MAP (mmHg)	62 ± 13	63 ± 15	60 ± 8	63 ± 13	61 ± 61
Hemoglobin (g/dl)	10.1 ± 1.6	10.2 ± 1.6	9.8 ± 1.6	10.4 ± 1.6	9.7 ± 1.5
NE dose (μg/kg/minute)	0.2 (0.06 to 0.34)	0.15 (0.04 to 0.475)	0.225 (0.08 to 0.32)	0.18 (0.07 to 0.5)	0.2 (0.1 to 0.3)
Vasopressin (u/minute)	0.04 (0.03 to 0.04)	0.04 (0.03 to 0.04)	0.04 (0.03 to 0.04)	0.04 (0.03 to 0.04)	0.04 (0.03 to 0.04)
ScvO_2_ (%)	72 ± 11	70 ± 13	75 ± 8	72 ± 11	72 ± 12
PaO_2_/FiO_2_ (mmHg)	195 (128 to 290)	247 (153 to 310)*	163.5 (113 to 199)*	248.5 (76 to 300)	175 (124 to 260)
pCO_2_ (mmHg)	40 ± 12	38 ± 9*	45 ± 14*	38 ± 11	43 ± 2
pH	7.29 ± 0.1	7.3 ± 0.1	7.27 ± 0.1	7.3 ± 0.1	7.29 ± 0.1
Lactate (mmol/l)	3 ± 2.8	2.4 ± 0.4*	4.2 ± 0.6*	1.45 (1 to 3.78)	2.4 (1.4 to 4.2)
Creatinine (mg/dl)	1.9 ± 1	2.1 ± 1.2	1.6 ± 0.7	1.84 ± 0.9	2 ± 1.2
Troponin T (ng/ml)	0.03 (0.01 to 0.16)	0.03 (0.01 to 0.2)	0.03 (0.01 to 0.12)	0.025 (0.01 to 0.2)	0.03 (0.01 to 0.1)
Clinical					
Respiratory issues	18 (30%)	12	6	7	11
Coronary artery disease	8 (13%)	7	1	3	5
Chronic renal failure	7 (12%)	5	2	4	3
Acute kidney injury	25 (42%)	17	8	12	13
Mechanical ventilation	39 (65%)	21*	18*	15*	24*

Data presented as *n* (%), mean ± standard deviation or median (interquartile range). *P <0.05 by analysis of variance. MAP, mean arterial pressure; NE, noradrenaline; pCO_2_, partial pressure of carbon dioxide; PaO_2_/FiO_2_, arterial partial pressure of oxygen/fraction of inspired oxygen ratio; ScvO_2_, central venous oxygen saturation; SOFA, Sequential Organ Failure Assessment.

### Echocardiographic analysis

**Figure 2:**
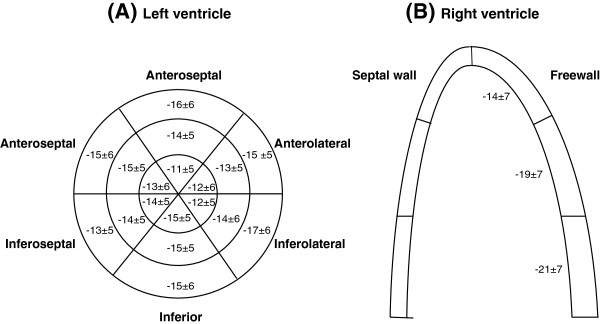
**Left and right ventricle segmental longitudinal strain values. (A)** Graphical representation of left ventricle segmental longitudinal strain with three concentric circles representing apex (inner circle), mid and base (outer circle). **(B)** Graphical representation of right ventricle segmental free wall longitudinal strain. Data presented as mean ± standard deviation.

There was no difference seen between survivors and nonsurvivors in any standard echocardiography measure of ventricle size or function at 30 days or 6 months, or in their peak systolic pulmonary artery pressures (Tables 2 and 3 and Figure 2). There was a significant difference in RV free wall strain between survivors and nonsurvivors at 6 months (−19% ± 5 vs. −16% ± 6, *P* = 0.02). There was no difference in survivors’ LV GLS or GLS rate compared with nonsurvivors.

**Table 2:** Echocardiography data at baseline and compared for survival at 30 days and 6 months

**Characteristic**	**Baseline**	**30-day mortality**		**6-month mortality**	
		**Survivors**	**Nonsurvivors**	**Survivors**	**Nonsurvivors**
Structure					
RV basal length (mm)	39 ± 7	40 ± 8	38 ± 5	40 ± 8	38 ± 6
RV mid length (mm)	33 ± 7	33 ± 7	33 ± 5	34 ± 7	33 ± 6
RV longitudinal length (mm)	75 ± 9	75 ± 10	74 ± 8	76 ± 10	73 ± 8
LV diastolic diameter (mm)	47 ± 5	47 ± 6	48 ± 4	47 ± 1	48 ± 5
LV systolic diameter (mm)	32 ± 7	33 ± 7	30 ± 5	33 ± 7	31 ± 6
Ventricular function					
RV FAC (%)	40 ± 10	40 ± 10	39 ± 10	40 ± 8	39 ± 11
Lateral tricuspid annular TDI velocity (cm/second)	15 ± 5	14 ± 5	17 ± 5	13 ± 4	16 ± 6
Cardiac index (l/minute/m^2^)	3.5 ± 1.5	3.5 ± 1.7	3.5 ± 1.2	3.5 ± 1.4	3.4 ± 1.7
SVI (cm^3^/m^2^)	37 ± 15	37 ± 16	38 ± 13	39 ± 14	35 ± 16
LV ejection fraction (%)	57 ± 16	56 ± 17	60 ± 13	55 ± 15	59 ± 16
Other parameters					
Echo assessed SPAP (mmHg)	42 ± 15	41 ± 14	44 ± 17	39 ± 14	46 ± 15

Data presented as mean ± standard deviation. FAC, fractional area change; LV, left ventricle; RV, right ventricle; TDI, tissue Doppler imaging; SVI, stroke volume index; SPAP, peak systolic pulmonary artery pressure.

**Table 3:** Baseline ventricular longitudinal strain with comparison for survival at 30 days and 6 months

**Characteristic**	**Baseline**	**30-day mortality**		**6-month mortality**	
		**Survivors**	**Nonsurvivors**	**Survivors**	**Nonsurvivors**
RV free wall strain (%)	−17.7 ± 5.5	−18.1 ± 5.4	−16.9 ± 5.6	−19.3 ± 4.9*	−16.0 ± 5.7*
RV free wall strain rate (1/second)	−1.14 ± 0.4	−1.14 ± 0.33	−1.14 ± 0.4	−1.19 ± 0.3	−1.09 ± 0.4
LV GLS (%)	−14.1 ± 4.2	−13.92 ± 4.2	−14.6 ± 4.3	−14 ± 4	−14.28 ± 4.6
LV GLS rate (1/second)	−0.89 ± 0.3	−0.86 ± 0.2	−0.96 ± 0.3	−0.86 ± 0.2	−0.93 ± 0.3

Data presented as mean ± standard deviation. *P <0.05 by analysis of variance. GLS, global longitudinal strain; LV, left ventricle; RV, right ventricle.

The incidence of myocardial dysfunction was different based on the method of assessment (Table 4). Based on conventional assessment, 19 patients (32%) had RV dysfunction, 20 patients (33%) had LV dysfunction and 10 patients (17%) had both LV and RV dysfunction. Based on strain analysis, 43 patients (72%) had RV dysfunction, 36 patients (69%) had LV dysfunction and 30 patients (50%) had both LV and RV strain dysfunction. When subgroups were created based on severity of dysfunction, strain function analysis also revealed a greater portion of patients with severe RV or LV dysfunction.

**Table 4:** Univariate analysis of systolic dysfunction and association with 30-day and 6-month mortality

		**Baseline**	**30-day mortality**			**6-month mortality**		
			**Survivors**	**Nonsurvivors**	***P *** **value**	**Survivors**	**Nonsurvivors**	***P *** **value**
**Strain analysis**								
RV free wall strain	RV dysfunction	43/60 (72%)			0.33			**0.001**
	• < −21	17	13	4		**12**	**5**	
	• –13 to −21	31	21	10		**18**	**13**	
	• > −13	12	6	6		**1**	**11**	
GLS	LV dysfunction	36/52 (69%)			0.40			0.44
	• < −17	16	9	7		7	9	
	• –10 to −17	25	19	6		16	9	
	• > −10	11	8	3		6	5	
**Standard echocardiographic analysis**								
RV dysfunction		19/60 (32%)			0.26			0.17
Mild		12	6	6		3	9	
Moderate		4	3	1		3	1	
Severe		3	3	0		2	1	
LV dysfunction		20/60 (33%)			0.50			0.55
Mild (EF = 45 to 55%)		8	5	3		4	4	
Moderate (EF = 35 to 45%)		8	7	1		6	2	
Severe (EF < 35%)		4	3	1		2	2	

Data presented as *n* (%). Strain is a measure of myocardial deformation and is described in negative values; greater negative numbers indicate better function. EF, ejection fraction; GLS, global longitudinal strain; LV, left ventricle; RV, right ventricle.

**Figure 3:**
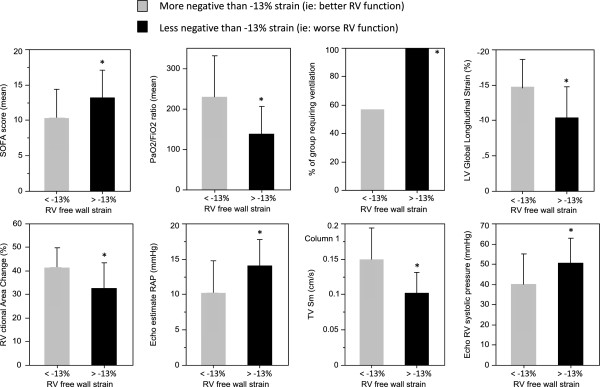
**Association of right ventricle free wall systolic strain with clinical and echocardiography parameters of disease severity and right ventricular dysfunction.** Error bars ± standard deviation. LV, left ventricle; PaO_2_/FiO_2_, arterial partial pressure of oxygen/fraction of inspired oxygen ratio; RAP, right atrial pressure; RV, right ventricle; SOFA, Sequential Organ Failure Assessment; TV Sm, Tricuspid valve systolic motion velocity.

**Figure 4:**
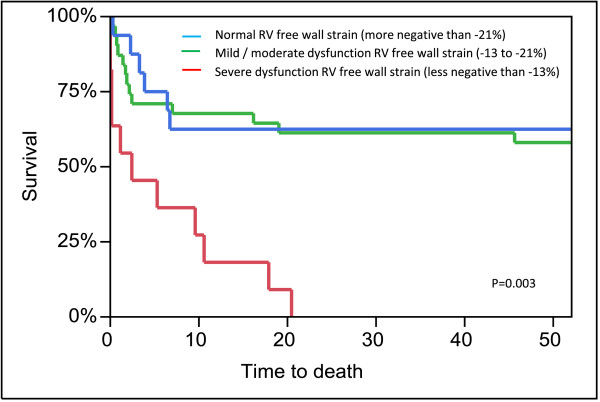
**Kaplan–Meier 1-year survival curves based on right ventricle free wall strain.** RV, right ventricle.

Analysis of variance of the association between 6-month mortality and the RV strain dysfunction subgroups was significant (*P* <0.001). Separate analysis within these groups exposed those patients with severe RV strain dysfunction as having the statistically significant association (Table 5). Multivariate analysis (Table 6) showed that severe RV free wall strain dysfunction remained an independent predictor of outcome at 6 months, accounting for mechanical ventilation (*P* = 0.03). This subgroup was also associated with a greater severity of disease (SOFA score), lower arterial partial pressure of oxygen/fraction of inspired oxygen ratios, mechanical ventilation, worse LV GLS, reduced RV FAC, higher echo-based right atrial pressures, lower tricuspid velocity, and higher echo-based peak systolic pulmonary artery pressure (Figure 3). Furthermore, there was a tendency towards higher levels of lactate. There was no association with RV dimensions. By comparison, RV systolic functional assessment by FAC was only associated with reduced LV GLS, and increased echo-based right atrial and RV systolic pressures. Kaplan–Meier curves show severe RV free wall longitudinal strain dysfunction was associated with 1-year mortality (*P* <0.001) due to all patients in this subgroup dying before 6 months (Figure 4). Those with mild/moderate RV strain dysfunction and normal RV strain function had similar 1-year survival estimates (57.1% and 54.9% respectively).

**Table 5:** Odds ratios for subsets of right ventricular free wall strain versus 6-month mortality

**Subgroup**	**Odds ratio**	**95% confidence interval**	***P *** **value**
Severe vs. mild/moderate (strain > −13 vs. −13 to −21)	15.23	2.5 to 296.27	**0.002**
Severe vs. normal (strain > −13 vs. < −21)	26.4	3.7 to 553.78	**<0.001**
Mild/moderate vs. normal (strain –13 to –21 vs. > −21)	1.73	0.5 to 6.56	0.4

**Table 6:** Multivariate analysis of severe right ventricle strain dysfunction and mechanical ventilation with 6-month mortality

	**6-month mortality**		
**Parameter**	**Odds ratio**	**95% confidence interval**	***P *** **value**
Severe right ventricle strain dysfunction	11.9	1.9 to 232	**0.03**
Mechanical ventilation	3.0	0.88 to 11.2	0.09

Pulmonary hypertension was not an exclusion criterion, but no patient had a formal diagnosis at time of enrollment. Fifteen patients had echocardiograms performed in the preceding 6 months to admission and four of these patients had peak systolic pulmonary artery pressure estimation >36 mmHg, considered raised pulmonary pressure by the American Society of Echocardiography [16]. Although no significant RV dysfunction was reported, there may have been unrecognized prior RV strain dysfunction. Excluding these patients from the analysis did not alter the relationship between 6-month mortality and RV free wall strain dysfunction.

### Measurement variability

Blinded interrater variability for STE analysis was assessed by JNS on a random 10% subgroup. Bland–Altman analysis demonstrated good intraobserver and interobserver agreement. The interobserver and intraobserver mean difference (±standard deviation) were respectively: RV free wall longitudinal strain, –2 (±1.2) and −1.4 (±0.9); RV free wall longitudinal strain rate, −0.3 (±0.1) and −0.1 (±0.05); LV GLS, −0.9 (±0.9%) and −0.8 (±0.5); and LV GLS rate, −0.1 (±0.05) and −0.1 (±0.05).

## Discussion

In this observational cohort study of 60 patients with severe sepsis or septic shock we demonstrated: frequent biventricular systolic dysfunction, occasionally severe, occurring within 24 hours of diagnosis; that STE is a more sensitive method of assessing systolic dysfunction than conventional echocardiography; and that severe RV dysfunction assessed by STE (RV free wall strain) is associated with a worse prognosis.

The use of STE in the noncritically ill population is increasing because most modern high-end echocardiography machines have the software capability. These advanced machines are becoming increasingly available in the ICU and it is suggested that STE may unmask systolic dysfunction not seen by standard echocardiographic assessment [11]. Indeed, a greater portion of the patients in our study were identified as having systolic dysfunction of both the RV and LV when assessed by STE as compared with conventional echocardiography. STE assessment of the LV GLS adds prognostic value in heart failure [22] and myocardial ischemia [20], and RV free wall strain analysis in pulmonary vascular disease trumps all other measures of RV function in the independent prediction of clinical deterioration and mortality and may help guide therapy [21,23-25]. In our septic population, RV free wall strain was the only parameter associated with mortality.

STE is dependent on adequate image quality, and studies in the noncritically ill report a 7 to 9% suboptimal image quality for STE analysis [18,20]. Imaging in the critically ill can be difficult and 13% of our patients were excluded due to poor image quality; however, STE was still feasible in the majority of our patients with adequate images. However, the difficulty in imaging analysis may explain why the interobserver variability in our study was slightly higher compared with others [18].

Myocardial dysfunction in sepsis is caused by a variety of factors, including direct effect by the infectious process (inflammatory mediators, bacterial toxins, and/or myocardial mitochondrial dysfunction), decreased myocardial perfusion, interventricular dependence and raised pulmonary pressures from acute lung injury, hypoxia, hypercarbia and atelectasis. Evaluation of myocardial contractile function by echocardiography is challenging, particularly for the RV due to its complex geometry, which makes volumetric assessment difficult. Several studies have found RV systolic dysfunction early in the course of sepsis to be associated with increased mortality [26-28]. However, other studies have found no significant difference between survivors and nonsurvivors [2,29]. A similar debate exists concerning LV dysfunction and outcomes [30-33]. A recent meta-analysis failed to find any evidence of differences in RV or LV function related to mortality [34].

The association between 6-month mortality and severe RV free wall strain dysfunction highlights the importance of RV function analysis in the prognosis of the critically ill patient, and this supports studies in other populations such as patients with acute respiratory distress syndrome [35]. All of the patients in our study with severe RV strain dysfunction died within 6 months of admission to the ICU with severe sepsis, potentially due to being sicker on admission (higher SOFA scores), being more likely to be on mechanical ventilation, or having worse gas exchange, worse LV GLS function and higher echo-based RV systolic pulmonary pressure estimation than patients without severe RV dysfunction. A myriad of factors are at play, and the RV can be affected by all of them – RV dysfunction is therefore likely to be a marker of disease severity as much as being a factor behind the association with poor outcomes. However, early recognition of RV dysfunction may help in the care of the critically ill patient with sepsis and may place emphasis on limiting factors that are potentially involved, such as fluid overload, high positive end-expiratory pressure levels, atelectasis, hypoxia or hypercarbia, amongst others.

### Limitations

This study is observational in nature and has a limited number of patients, and the imaging was not optimized for STE (for example, improved endocardial border definition, RV centric views, and so forth). One cannot exclude that weaker associations may be statistically significant in a cohort with a larger sample size. Repeated imaging and further STE analysis were not performed. Further dysfunction that would be seen by standard echo parameters may have occurred at a later stage. Larger, prospective studies with imaging focused for STE optimization and follow-up echocardiography STE analysis could be considered in future.

There are several drawbacks to current STE analysis that limit its clinical utility in the ICU at this time: STE requires adequate image quality, which can be challenging particularly in the mechanically ventilated patient (10 of the 14 patients excluded due to inadequate image quality), STE is time consuming to perform, and normal values have been difficult to elucidate partly due to vendor differences in the software algorithms [19]. Our cutoff values between normality, mild and moderate abnormality and severe abnormality are similar to recent studies on large populations of both normal controls [18,19] and patients with cardiac dysfunction [25]. With technology advancing and expert groups such as the American Society of Echocardiography and the European Association of Echocardiography calling for concordance on vendor STE software analysis, and as the use of STE becomes more widespread, perhaps strain will become a more standard measurement in the future [17].

## Conclusions

STE unmasks systolic dysfunction unrecognized with conventional echocardiography in patients with severe sepsis or septic shock. RV dysfunction assessed by strain appears to be correlated with worse late outcomes, especially if the dysfunction is severe. LV dysfunction assessed either by conventional imaging or STE does not appear to correlate with survival in sepsis.

## Key messages

● STE unmasks systolic dysfunction unrecognized with conventional echocardiography in patients with severe sepsis or septic shock.

● Severe right ventricular strain dysfunction is associated with worse prognosis.

● LV dysfunction assessed by standard echocardiography or STE is not associated with early or late outcome.

## Abbreviations

FAC: fractional area change; GLS: global longitudinal strain; LV: left ventricle; RV: right ventricle; SOFA: Sequential Organ Failure Assessment; STE: speckle tracking echocardiography.

## Competing interests

The authors declare that they have no competing interests.

## Authors’ contributions

SRO, JNP, MM, SG, JNS, GCK and JKO contributed to data acquisition and take responsibility for the integrity and accuracy of the data and analysis. SRO, JNP, GCK and JKO had access to the data, and contributed to study conception and design, statistical analysis and preparation of the manuscript. MM and SG contributed to study conception and design, and preparation of the manuscript. JNS contributed to data analysis and interpretation, and drafting of the manuscript. All authors read and approved the final manuscript.
